# Supplementary material for: Seasonal and successional dynamics of size-dependent plant demographic rates in a tropical dry forest
Source: PeerJ. 2020 Sep 14;8:e9636. doi: 10.7717/peerj.9636 (PMC7497611; doi:10.7717/peerj.9636)
Supplement: Table S1 [file peerj-08-9636-s001.docx]

**Table S1:** Successional (forest stand) age and category of each study plot.

| Forest stand age | Plot | Stand age in 2009 (yr) | Stand age in 2015 (yr) |
| --- | --- | --- | --- |
| Early stage | A | 3 | 9 |
|  | B | 3 | 9 |
|  | C | 5 | 11 |
| Intermediate stage | A | 20 | 26 |
|  | B | 18 | 24 |
|  | C | 19 | 25 |
| Old-growth stage | A | 60 | 66 |
|  | B | 55 | 61 |
|  | C | 65 | 71 |
