# Supplementary material for: Seasonal and successional dynamics of size-dependent plant demographic rates in a tropical dry forest
Source: PeerJ. 2020 Sep 14;8:e9636. doi: 10.7717/peerj.9636 (PMC7497611; doi:10.7717/peerj.9636)
Supplement: Table S2 — Significant P values (≤0.05) are indicated in boldface. The standard errors (SE), conditional R2 (R2c, both fixed and random effects), and the marginal R2 (R2m, fixed effects only) as well as the relative (%) difference between them (indicating the importance of random effects) are shown. [file peerj-08-9636-s002.docx]

| Fixed effects | | Plant density  *R^2^m* = 0.701; *R^2^c* = 0.992 (29%) | | | Species density  *R^2^m* = 0.735; *R^2^c* = 0.901 (26%) | | |
| --- | --- | --- | --- | --- | --- | --- | --- |
|  |  | Estimate | SE | *P*-value | Estimate | SE | *P*-value |
| Early stage | Dry | **-1515.33** | **204.65** | **0.01** | **-12.18** | **1.76** | **2.62** × 10**^-4^** |
|  | Dry : Year | **172.95** | **8.01** | **2.22 × 10^-37^** | **1.40** | **0.23** | **3.21** × 10**^-8^** |
|  | Wet | **95.33** | **44.09** | **0.03** | 1.44 | 1.27 | 0.26 |
|  | Wet : Year | 3.71 | 11.32 | 0.74 | -0.14 | 0.33 | 0.66 |
| Intermediate stage | Dry | -198.0 | 254.85 | 0.48 | -3.04 | 1.43 | 0.08 |
|  | Dry : Year | -4.38 | 8.01 | 0.59 | 0.17 | 0.23 | 0.46 |
|  | Wet | -58.89 | 44.09 | 0.19 | -0.20 | 1.27 | 0.88 |
|  | Wet : Year | 14.29 | 11.32 | 0.21 | 0.06 | 0.33 | 0.86 |
| Advanced stage | Dry (Intercept) | **1527.56** | **193.06** | **0.01** | **16.42** | **1.26** | **5.48 × 10^-4^** |
|  | Dry : Year | 2.76 | 5.66 | 0.63 | 0.18 | 0.16 | 0.27 |
|  | Wet | 20.89 | 31.18 | 0.50 | 0.07 | 0.90 | 0.94 |
|  | Wet : Year | -5.33 | 8.01 | 0.51 | -0.02 | 0.23 | 0.93 |
