# Supplementary material for: Seasonal and successional dynamics of size-dependent plant demographic rates in a tropical dry forest
Source: PeerJ. 2020 Sep 14;8:e9636. doi: 10.7717/peerj.9636 (PMC7497611; doi:10.7717/peerj.9636)
Supplement: Table S3 — Significant P values (≤0.05) are indicated in boldface. The standard errors (SE), conditional R2 (R2c, both fixed and random effects), and the marginal R2 (R2m, fixed effects only) as well as the relative (%) difference between them (indicating the importance of random effects) are shown. [file peerj-08-9636-s003.docx]

| Fixed effects | | Plant density  *R^2^m* = 0.557; *R^2^c* = 0.991 (44%) | | | Species density  *R^2^m* = 0.336; *R^2^c* = 0.983 (66%) | | |
| --- | --- | --- | --- | --- | --- | --- | --- |
|  |  | Estimate | SE | *P*-value | Estimate | SE | *P*-value |
| Early stage | Dry | 5983.7 | 2139.2 | 0.10 | **-17.7** | **5.2** | **0.03** |
|  | Dry : Year | -81.6 | 58.8 | 0.17 | **1.4** | **0.3** | **4.59^-7^** |
|  | Wet | -103.7 | 323.9 | 0.75 | -0.2 | 1.4 | 0.87 |
|  | Wet : Year | 6.3 | 83.2 | 0.94 | 0.3 | 0.4 | 0.42 |
| Intermediate stage | Dry | 1298.5 | 548.2 | 0.08 | -13.8 | 7.7 | 0.15 |
|  | Dry : Year | 53.3 | 58.8 | 0.37 | **0.6** | **0.3** | **0.01** |
|  | Wet | -59.3 | 323.9 | 0.86 | -0.2 | 1.4 | 0.91 |
|  | Wet : Year | 17.5 | 83.2 | 0.83 | 0.1 | 0.4 | 0.83 |
| Advanced stage | Dry (Intercept) | **7443.7** | **240.5** | **1.49^-7^** | **43.4** | **4.4** | **0.01** |
|  | Dry : Year | **-298.7** | **41.6** | **1.89^-10^** | **-0.9** | **0.2** | **5.36^-6^** |
|  | Wet | -132.6 | 229.0 | 0.56 | 0.1 | 1.0 | 0.93 |
|  | Wet : Year | 1.9 | 58.8 | 0.97 | -0.1 | 0.3 | 0.82 |
