# Supplementary material for: Seasonal and successional dynamics of size-dependent plant demographic rates in a tropical dry forest
Source: PeerJ. 2020 Sep 14;8:e9636. doi: 10.7717/peerj.9636 (PMC7497611; doi:10.7717/peerj.9636)
Supplement: Table S4 — Significant P values (≤0.05) are indicated in boldface. The standard errors (SE), conditional R2 (R2c, both fixed and random effects), and the marginal R2 (R2m, fixed effects only) as well as the relative (%) difference between them (indicating the importance of random effects) are shown. [file peerj-08-9636-s004.docx]

| Fixed effects | | Recruitment rate  *R^2^m* =0.682; *R^2^c* = 0.701 (3%) | | | | Species gain rate  *R^2^m* = 0.333; *R^2^c* = 0.353 (6%) | | |
| --- | --- | --- | --- | --- | --- | --- | --- | --- |
|  |  | Estimate | SE | | *P*-value | Estimate | SE | *P*-value |
| Early stage | **Dry** | -0.001 | 0.07 | 0.99 | | -0.021 | 0.07 | 0.77 |
|  | **Dry : Year** | 0.015 | 0.02 | 0.36 | | 0.014 | 0.02 | 0.44 |
|  | **Wet** | **0.614** | **0.09** | **9.64 × 10^-10^** | | **0.348** | **0.10** | **7.04 × 10^-4^** |
|  | **Wet : Year** | **-0.105** | **0.02** | **1.85 × 10^-5^** | | **-0.063** | **0.03** | **0.015** |
| Intermediate stage | **Dry** | 0.012 | 0.06 | 0.85 | | 0.015 | 0.07 | 0.83 |
|  | **Dry : Year** | -0.001 | 0.02 | 0.96 | | 0.000 | 0.02 | 0.99 |
|  | **Wet** | -0.011 | 0.09 | 0.91 | | -0.013 | 0.10 | 0.90 |
|  | **Wet : Year** | 0.004 | 0.02 | 0.87 | | 0.001 | 0.03 | 0.98 |
| Advanced stage | **Dry (Intercept)** | 0.009 | 0.05 | 0.85 | | 0.002 | 0.05 | 0.96 |
|  | **Dry : Year** | -0.001 | 0.01 | 0.94 | | 0.001 | 0.01 | 0.94 |
|  | **Wet** | 0.008 | 0.06 | 0.91 | | 0.006 | 0.07 | 0.93 |
|  | **Wet : Year** | -0.0002 | 0.02 | 0.99 | | -0.002 | 0.02 | 0.92 |
