# Supplementary material for: Seasonal and successional dynamics of size-dependent plant demographic rates in a tropical dry forest
Source: PeerJ. 2020 Sep 14;8:e9636. doi: 10.7717/peerj.9636 (PMC7497611; doi:10.7717/peerj.9636)
Supplement: Table S5 — Significant P values (≤0.05) are indicated in boldface. The standard errors (SE), conditional R2 (R2c, both fixed and random effects), and the marginal R2 (R2m, fixed effects only) as well as the relative (%) difference between them (indicating the importance of random effects) are shown. [file peerj-08-9636-s005.docx]

| Fixed effects | | Recruitment rate  *R^2^m* =0.143; *R^2^c* = 0.188 (24%) | | | Species gain rate  *R^2^m* = 0.209; *R^2^c* = 0.209 (0%) | | |
| --- | --- | --- | --- | --- | --- | --- | --- |
|  |  | Estimate | SE | *P*-value | Estimate | SE | *P*-value |
| Early stage | **Dry** | 0.001 | 0.014 | 0.94 | -0.004 | 0.022 | 0.85 |
|  | **Dry : Year** | 0.001 | 0.003 | 0.71 | 0.002 | 0.006 | 0.75 |
|  | **Wet** | 0.017 | 0.019 | 0.35 | 0.015 | 0.030 | 0.62 |
|  | **Wet : Year** | -0.004 | 0.005 | 0.38 | 0.004 | 0.008 | 0.59 |
| Intermediate stage | **Dry** | 0.001 | 0.013 | 0.95 | -0.003 | 0.021 | 0.91 |
|  | **Dry : Year** | 0.001 | 0.003 | 0.87 | 0.002 | 0.006 | 0.69 |
|  | **Wet** | 0.004 | 0.019 | 0.82 | -0.003 | 0.030 | 0.93 |
|  | **Wet : Year** | -0.001 | 0.005 | 0.90 | -0.001 | 0.008 | 0.87 |
| Advanced stage | **Dry (Intercept)** | 0.002 | 0.009 | 0.80 | 0.001 | 0.015 | 0.92 |
|  | **Dry : Year** | 1.34 × 10^-4^ | 0.002 | 0.96 | -1.57 × 10^-4^ | 0.004 | 0.97 |
|  | **Wet** | 0.005 | 0.013 | 0.68 | 0.013 | 0.021 | 0.55 |
|  | **Wet : Year** | 0.002 | 0.003 | 0.64 | -0.001 | 0.006 | 0.86 |
