# Supplementary material for: Seasonal and successional dynamics of size-dependent plant demographic rates in a tropical dry forest
Source: PeerJ. 2020 Sep 14;8:e9636. doi: 10.7717/peerj.9636 (PMC7497611; doi:10.7717/peerj.9636)
Supplement: Table S6 — Significant P values (≤0.05) are indicated in boldface. The standard errors (SE), conditional R2 (R2c, both fixed and random effects), and the marginal R2 (R2m, fixed effects only) as well as the relative (%) difference between them (indicating the importance of random effects) are shown. [file peerj-08-9636-s006.docx]

| Fixed effects | | Mortality rate  *R^2^m* =0.331; *R^2^c* = 0.385 (14%) | | | Species loss rate  *R^2^m* = 0.086; *R^2^c* = 0.133 (35%) | | |
| --- | --- | --- | --- | --- | --- | --- | --- |
|  |  | Estimate | SE | *P*-value | Estimate | SE | *P*-value |
| Early stage | Dry | -0.006 | 0.009 | 0.523 | 1.62 × 10^-17^ | 0.012 | 1.00 |
|  | Dry : Year | -1.17 × 10^-4^ | 0.002 | 0.960 | -4.68 × 10^-18^ | 0.003 | 1.00 |
|  | Wet | 7.35 × 10^-5^ | 0.013 | 0.995 | -1.98 × 10-^3^ | 0.017 | 0.91 |
|  | Wet : Year | -6.44 × 10^-5^ | 0.003 | 0.984 | -8.50 × 10^-4^ | 0.004 | 0.85 |
| Intermediate stage | Dry | 0.006 | 0.010 | 0.547 | -6.99 × 10^-3^ | 0.013 | 0.59 |
|  | Dry : Year | 3.89 × 10^-5^ | 0.002 | 0.987 | 2.99 × 10^-3^ | 0.003 | 0.34 |
|  | Wet | **0.034** | **0.013** | **0.010** | 2.81 × 10^-2^ | 0.017 | 0.11 |
|  | Wet : Year | **-0.007** | **0.003** | **0.031** | -7.46 × 10^-3^ | 0.004 | 0.10 |
| Advanced stage | Dry (Intercept) | 0.006 | 0.006 | 0.370 | -1.12 × 10^-17^ | 0.009 | 1.00 |
|  | Dry : Year | 1.17× 10^-4^ | 0.002 | 0.943 | 3.38 × 10^-18^ | 0.002 | 1.00 |
|  | Wet | -0.003 | 0.009 | 0.717 | 1.98 × 10^-3^ | 0.012 | 0.87 |
|  | Wet : Year | 0.002 | 0.002 | 0.432 | 8.50 × 10^-4^ | 0.003 | 0.79 |
