# Supplementary material for: Seasonal and successional dynamics of size-dependent plant demographic rates in a tropical dry forest
Source: PeerJ. 2020 Sep 14;8:e9636. doi: 10.7717/peerj.9636 (PMC7497611; doi:10.7717/peerj.9636)
Supplement: Table S7 — Significant P values (≤0.05) are indicated in boldface. The standard errors (SE), conditional R2 (R2c, both fixed and random effects), and the marginal R2 (R2m, fixed effects only) as well as the relative (%) difference between them (indicating the importance of random effects) are shown. [file peerj-08-9636-s007.docx]

| Fixed effects | | Mortality rate  *R^2^m* =0.177; *R^2^c* = 0.237 (25%) | | | Species loss rate  *R^2^m* = 0.042; *R^2^c* = 0.061 (31%) | | | |
| --- | --- | --- | --- | --- | --- | --- | --- | --- |
|  |  | Estimate | SE | *P*-value | Estimate | SE | | *P*-value |
| Early stage | Dry | -0.006 | 0.011 | 0.58 | 0.002 | | 0.016 | 0.93 |
|  | Dry : Year | -0.001 | 0.003 | 0.82 | -0.002 | | 0.004 | 0.58 |
|  | Wet | 0.011 | 0.015 | 0.47 | 0.010 | | 0.023 | 0.68 |
|  | Wet : Year | -2.14 × 10^-4^ | 0.004 | 0.96 | -0.002 | | 0.006 | 0.76 |
| Intermediate stage | Dry | 1.17 × 10^-4^ | 0.011 | 0.99 | 0.007 | | 0.016 | 0.67 |
|  | Dry : Year | -0.002 | 0.003 | 0.48 | -0.003 | | 0.004 | 0.53 |
|  | Wet | 3.85 × 10^-4^ | 0.015 | 0.98 | -0.017 | | 0.023 | 0.47 |
|  | Wet : Year | 0.002 | 0.004 | 0.63 | 0.004 | | 0.006 | 0.52 |
| Advanced stage | Dry (Intercept) | **0.020** | **0.008** | **0.01** | 0.009 | | 0.012 | 0.44 |
|  | Dry : Year | -0.001 | 0.002 | 0.79 | 0.002 | | 0.003 | 0.53 |
|  | Wet | 0.008 | 0.011 | 0.46 | 0.009 | | 0.016 | 0.60 |
|  | Wet : Year | -0.001 | 0.003 | 0.79 | -0.003 | | 0.004 | 0.53 |
